# Stress-induced inhibition of translation independently of eIF2α phosphorylation

**DOI:** 10.1242/jcs.176545

**Published:** 2015-12-01

**Authors:** Jon Halvor Jonsrud Knutsen, Gro Elise Rødland, Cathrine Arnason Bøe, Tine Weise Håland, Per Sunnerhagen, Beáta Grallert, Erik Boye

**Affiliations:** 1 Department of Radiation Biology, Institute for Cancer Research, Oslo University Hospital, Oslo, Norway; 2 Department of Chemistry & Molecular Biology, University of Gothenburg, Gothenburg, Sweden

**Keywords:** eIF2α, Stress, Translation, Ultraviolet light, *SPBC4B4.04*, EIF2S1, SUI2

## Abstract

Exposure of fission yeast cells to ultraviolet (UV) light leads to inhibition of translation and phosphorylation of the eukaryotic initiation factor-2α (eIF2α). This phosphorylation is a common response to stress in all eukaryotes. It leads to inhibition of translation at the initiation stage and is thought to be the main reason why stressed cells dramatically reduce protein synthesis. Phosphorylation of eIF2α has been taken as a readout for downregulation of translation, but the role of eIF2α phosphorylation in the downregulation of general translation has not been much investigated. We show here that UV-induced global inhibition of translation in fission yeast cells is independent of eIF2α phosphorylation and the eIF2α kinase general control nonderepressible-2 protein (Gcn2). Also, in budding yeast and mammalian cells, the UV-induced translational depression is largely independent of GCN2 and eIF2α phosphorylation. Furthermore, exposure of fission yeast cells to oxidative stress generated by hydrogen peroxide induced an inhibition of translation that is also independent of Gcn2 and of eIF2α phosphorylation. Our findings show that stress-induced translational inhibition occurs through an unknown mechanism that is likely to be conserved through evolution.

## Abstract (precis)

**Summary:** In contrast to textbook knowledge, the phosphorylation of translation initiation factor eIF2α is not required for UV-induced inhibition of protein synthesis, which we show in three different cell types.

## INTRODUCTION

All types of cells activate mechanisms to regulate gene expression after exposure to environmental stress, and to a large extent, at the level of translation. These stress responses enable the cells to cope with and adapt to stressful situations such as starvation, oxidative damage, osmotic stress and DNA damage. Translation can be regulated at all stages of the process, including initiation, elongation and termination, but control of translation in eukaryotes occurs mainly at the initiation step (reviewed in Sonenberg and Hinnebusch, 2009). It is well documented that even slight misregulation of translation seriously affects human health, leading to cancer (Castilho et al., 2014; Loreni et al., 2014) and neurological diseases (Buffington et al., 2014; Wells, 2012), underscoring the importance of characterizing the mechanisms of translational regulation.

A central mechanism for the regulation of translation is mediated by phosphorylation of the α subunit (encoded by *EIF2S1* in mammals, *SUI2* in budding yeast and *SPBC4B4.04* in fission yeast) of the initiation factor eIF2. An early step in initiation involves the binding of the 40S ribosomal subunit to the ternary complex comprising eIF2, the initiator tRNA (Met-tRNA_i_^Met^) and GTP (for a detailed overview, see Aitken and Lorsch, 2012). Together with the 40S ribosomal subunit, the ternary complex and additional initiation factors bind to and scan along the mRNA until an initiation codon is reached. At this stage, the 60S ribosomal subunit joins the complex, the GTP that is associated with eIF2 is hydrolysed, eIF2–GDP is released and translation proceeds. To be reused in subsequent rounds of initiation, eIF2–GDP must be converted to eIF2–GTP by the guanine nucleotide exchange factor eIF2B. Phosphorylation of eIF2α on residue S52 (S51 in mammalian cells) leads to a stable (eIF2–GDP)–eIF2B interaction, which inhibits the GDP–GTP exchange and precludes liberation of active eIF2, thereby reducing initiation of translation.

Phosphorylation of eIF2α is executed by evolutionarily conserved kinases, of which four are known in mammalian cells (GCN2, PERK, PKR and HRI), three in fission yeast (Gcn2, Hri1 and Hri2) and one in budding yeast (Gcn2). Many different forms of stress results in phosphorylation of eIF2α in eukaryotic cells (Clemens, 2001; Sonenberg and Hinnebusch, 2009), including deprivation of important nutrients (amino acids, heme or glucose), exposure to heat, heavy metals, high salt levels, hypoxia, oxidizing agents, DNA-damaging agents or chemicals.

In several different types of cells, protein synthesis is inhibited after exposure to ultraviolet (UV) light, including rat fibroblasts (Iordanov et al., 1998), mouse embryonic fibroblasts (Deng et al., 2002; Fox et al., 2009), human cells (Wu et al., 2002), maize leaves (Casati and Walbot, 2004) and fission yeast (Krohn et al., 2008; Tvegård et al., 2007). This downregulation has been ascribed to Gcn2-dependent phosphorylation of eIF2α, which has been shown to occur after UV irradiation (Deng et al., 2002; Tvegård et al., 2007; Wu et al., 2002). Although eIF2α is phosphorylated after exposure to UV, it is unknown whether this phosphorylation is responsible for the inhibition of translation.

There is convincing evidence that eIF2α phosphorylation attenuates the translation of most mRNAs. However, the importance of this mechanism might have been overestimated and, in many cases, presumed to be the mechanism behind an observed inhibition of protein synthesis after stress simply because eIF2α is phosphorylated. There are a few published sets of data that indicate the presence of an inhibitory stress-induced mechanism operating independently of eIF2α, such as after oxidative stress in budding yeast (Shenton et al., 2006) and after endoplasmic reticulum stress in mammalian cells (Hamanaka et al., 2005). However, these findings have received little attention and have not affected the general consensus in the field.

In the present work, we have investigated the role of eIF2α and the Gcn2 kinase in mediating stress-induced translational inhibition. We have directly measured the translation rate in fission yeast, budding yeast and mammalian cells after UV irradiation and shown that the inhibition of protein synthesis is largely independent of eIF2α phosphorylation in all three cell types. Our data suggest that a hitherto unrecognized mechanism regulates translation after stress.

## RESULTS

### Measuring protein synthesis in fission yeast cells

**Fig. 1.**
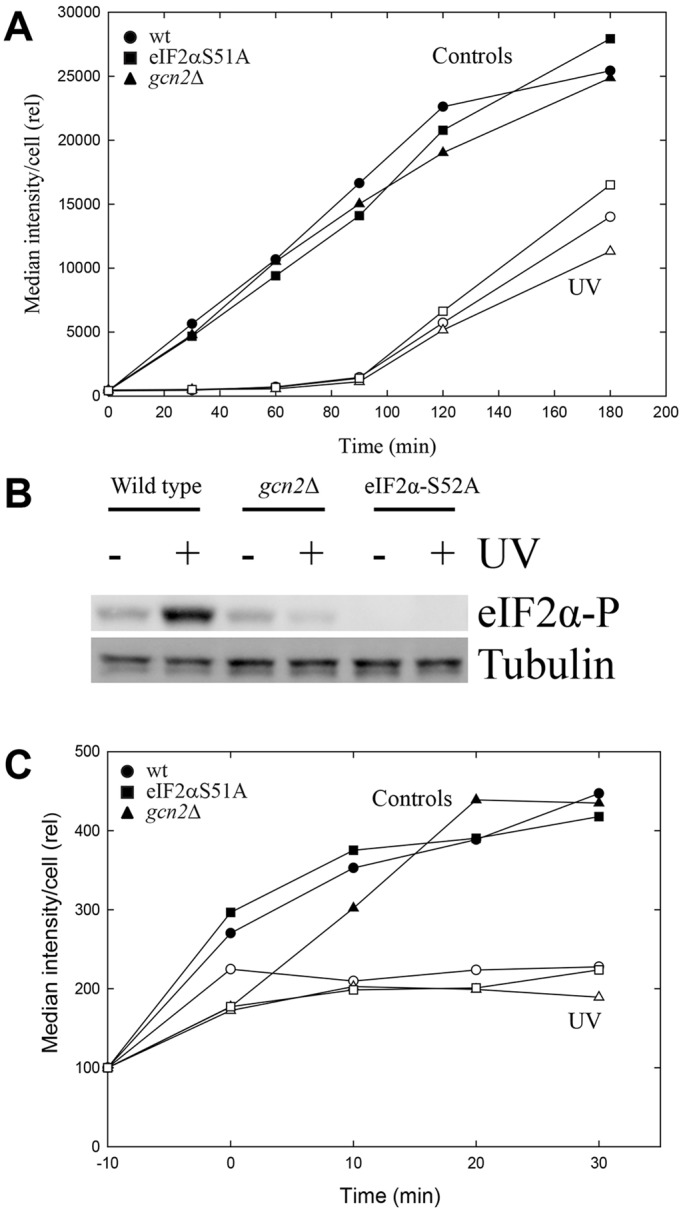
**Translation in *S. pombe* cells after UV irradiation.** (A) Incorporation of HPG into newly synthesized proteins in wild-type cells, *gcn2*Δ or eIF2αS52A mutants, when added immediately after exposure to UV (marked ‘UV’; open symbols) or without exposure (marked ‘Controls’; filled symbols). (B) Immunoblot of whole-cell extracts collected immediately after irradiation and probed with an antibody specific for phosphorylated eIF2α (eIF2α-P), with tubulin as a loading control. (C) As described for A, except that the cells were pre-incubated with HPG (added at time −10 min) to allow uptake before the medium was exchanged for one without HPG (at time 0, the time of irradiation; see text). Rel, relative.

We employed a non-radioactive, metabolic labelling assay to quantify protein synthesis. The method uses the noncanonical amino acid l-homopropargylglycine (HPG), which is incorporated into proteins in the place of methionine. The presence of HPG is detected by using chemoselective fluorescence-tagging by means of ‘click chemistry’ and has previously been used to label newly synthesized proteins in rat fibroblasts (Beatty and Tirrell, 2008) and rat hippocampal neurons (Dieterich et al., 2010) . We have adapted this method to measure protein synthesis in fission yeast cells (*Schizosaccharomyces pombe*). After adding HPG to exponentially growing cells, we observed an HPG-specific signal with fluorescence microscopy that increased continuously over time. The signal intensities from HPG-containing individual cells could be conveniently quantified using flow cytometry (Fig. 1A).

### UV-induced inhibition of translation is independent of Gcn2 and eIF2α phosphorylation

Previous studies have shown that exposure of fission yeast cells to UV leads to inhibition of translation, as measured by incorporation of radiolabelled amino acids *in vivo* and *in vitro* (Tvegård et al., 2007). Here, we used the novel translation assay to investigate the roles of Gcn2 and eIF2α phosphorylation in the UV-induced inhibition of protein synthesis. UV irradiation of wild-type cells caused an immediate and almost total inhibition of HPG incorporation that lasted for about 90 min (Fig. 1A), in addition to strong phosphorylation of eIF2α (Fig. 1B). Surprisingly, the same strong inhibition of translation was observed for the *gcn2*Δ mutant and the eIF2α-S52A mutant, which expresses a nonphosphorylatable version of eIF2α (Fig. 1A). Furthermore, the recovery of translation observed after 90 min of incubation was also independent of Gcn2 and eIF2α phosphorylation. No UV-induced phosphorylation of eIF2α could be detected in the two mutant strains, as expected (Fig. 1B).

**Fig. 2.**
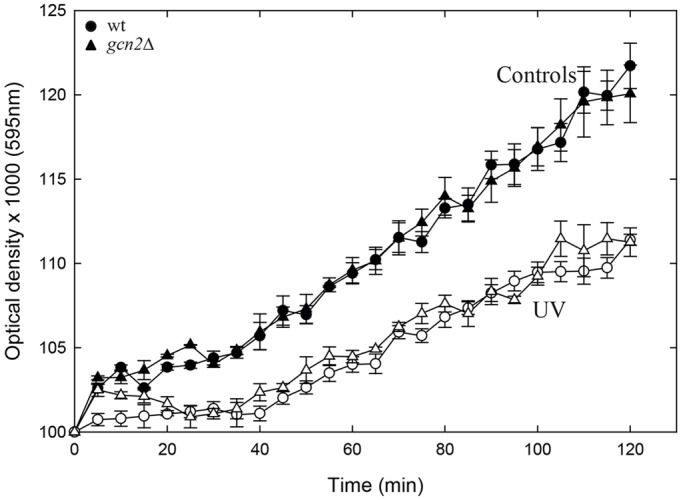
**Cell growth after UV exposure as measured using the optical density.** Wild-type or *gcn2*Δ mutant *S. pombe* cells were grown and irradiated in EMM medium at time 0, and the optical density at 595-nm light was recorded. Unirradiated control cells, filled symbols; UV-irradiated cells, open symbols. The mean and s.e.m. of the mean from three independent experiments are shown.

To exclude the possibility that the reduced incorporation of HPG after UV-irradiation was due to inhibition of import during or after UV irradiation, we incubated the cells with HPG and washed them with HPG-free medium to remove extracellular HPG before exposing them to UV light. Under these conditions, incorporation of HPG continued for 20 to 30 min (Fig. 1C), showing that the cells contained sufficient amounts of HPG to allow its incorporation into newly synthesized proteins. Moreover, also in these experiments, we observed an immediate inhibition of incorporation of HPG for all three strains after UV irradiation, showing that the inhibition observed above cannot be explained by reduced import of HPG after UV irradiation. In separate experiments, we verified that the UV dose used did not reduce the ability of HPG to function as a substrate in protein synthesis (J.H.J.K., unpublished data). As an alternative to the HPG measurements, we also monitored the optical density (OD) of the cultures, a parameter reflecting total mass growth that is independent of import of tracer amino acids or HPG. The optical density was measured after UV irradiation of both wild-type and *gcn2*Δ cells. The two strains showed the same reduction in growth (Fig. 2), supporting the conclusion that UV inhibits translation independently of Gcn2 and of eIF2α phosphorylation. These data argue that neither Gcn2 nor phosphorylation of eIF2α is required for the global downregulation of translation observed after UV irradiation of fission yeast cells.

### UV-induced inhibition of translation in budding yeast

**Fig. 3.**
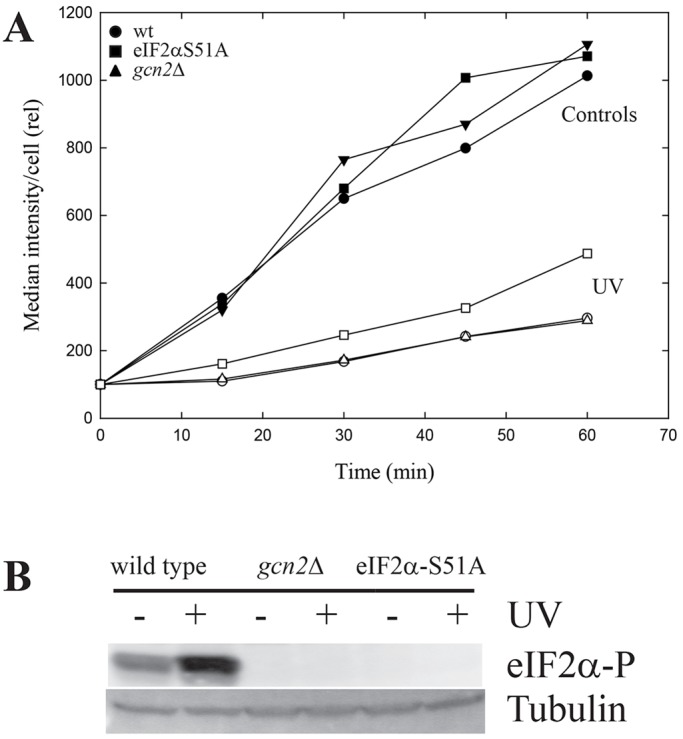
**Translation in *S. cerevisiae* cells after UV irradiation.** (A) Incorporation of HPG into newly synthesized proteins in wild-type cells, *gcn2*Δ mutant cells or cells containing the nonphosphorylatable version of eIF2α with (open symbols) or without (filled symbols) exposure to UV. (B) Immunoblot of whole-cell extracts taken immediately after irradiation and probed with an antibody specific for phosphorylated eIF2α (eIF2α-P), again with tubulin as a loading control.

Budding yeast (*Saccharomyces cerevisiae*) cells were investigated by using the same procedures as described for *S. pombe* cells. Because budding yeast are much less resistant to UV irradiation than fission yeast, the radiation dose was reduced to 220 J/m^2^. Again, UV exposure almost completely abolished protein synthesis in wild-type cells (Fig. 3A). Strong inhibition of protein synthesis was also observed in both mutant cells, but the UV-induced inhibition was consistently less in cells that carried a nonphosphorylatable version of eIF2α than in wild-type and *gcn2*Δ cells. The mutant cells did not phosphorylate eIF2α after exposure to UV (Fig. 3B), as expected. These data show that also in budding yeast protein, synthesis is dramatically reduced after UV irradiation through a mechanism that does not involve Gcn2 or eIF2α phosphorylation.

### UV-induced inhibition of translation in mammalian cells

**Fig. 4.**
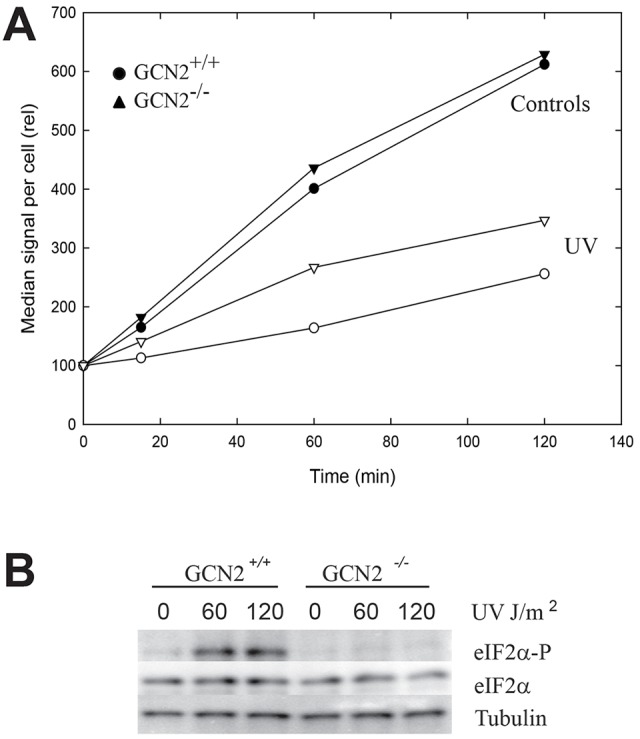
**Translation in MEF cells after UV irradiation.** (A) Incorporation of OPP into newly synthesized proteins in MEF GCN2^+/+^ and MEF GCN2^−/−^ cells with (open symbols) or without (filled symbols) UV irradiation. (B) Immunoblot of whole-cell extracts collected 30 min after the time of irradiation and probed with an antibody specific for phosphorylated eIF2α (eIF2α-P), with both total eIF2α and γ-tubulin as loading controls.

Analogous experiments to those above were performed with mammalian cells in culture. Mammalian cells do not incorporate exogenously added methionine (or HPG) unless starved for methionine before labelling. Such starvation has the unwanted effect of activating GCN2. Therefore, we instead employed the puromycin analogue O-propargyl-puromycin, OPP (Liu et al., 2012; Signer et al., 2014), which has been found to be efficiently incorporated into proteins in cells grown in complete medium and without starvation. In mouse embryo fibroblasts (MEFs), the exposure to 60 J/m^2^ of UV inhibited most of the incorporation (Fig. 4A). GCN2^−/−^ MEFs also displayed a strong inhibition of protein synthesis after UV irradiation, but the inhibition was slightly less than that observed for the GCN2^+/+^ MEFs (Fig. 4A). Immunoblotting verified that in GCN2^+/+^ MEF cells, eIF2α was phosphorylated, and no phosphorylation occurred in the knockout cells (Fig. 4B). These data suggest that the inhibition of protein synthesis induced by UV in mammalian cells is partly dependent of GCN2, but that there is another mechanism that is responsible for most of the inhibition.

### Translation after oxidative stress

**Fig. 5.**
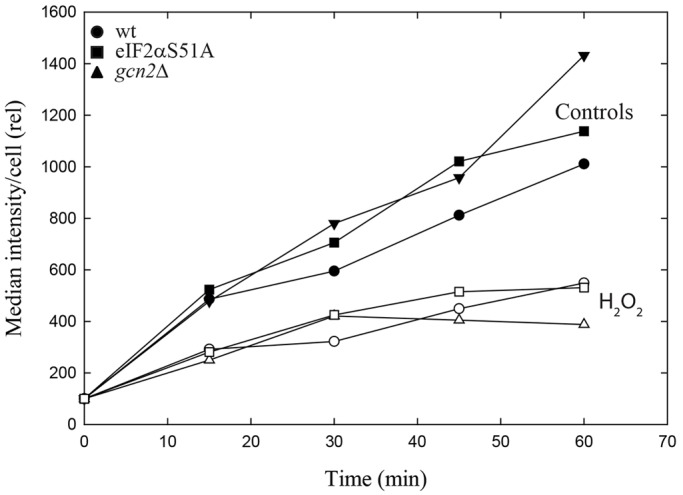
**Translation in *S. pombe* cells after oxidative stress.** Incorporation of HPG into newly synthesized proteins in wild-type cells, or *gcn2*Δ or eIF2αS52A mutant cells, after exposure to hydrogen peroxide (open symbols) or without exposure (filled symbols).

To determine whether the eIF2α-phosphorylation-independent downregulation of translation is specific for UV irradiation, we repeated the HPG-incorporation assay with *S. pombe* cells that had been exposed to hydrogen peroxide (H_2_O_2_), which leads to oxidative stress and Gcn2-dependent phosphorylation of eIF2α (Krohn et al., 2008). The rate of translation was much reduced for 60 min after exposure to H_2_O_2_ (Fig. 5). In the *gcn2*Δ and eIF2α-S52A mutants, translation was reduced to the same extent as that in wild-type cells. We conclude that translational inhibition after exposure to hydrogen peroxide is mainly independent of GCN2 and of eIF2α phosphorylation.

### What is the mechanism of inhibition?

**Fig. 6.**
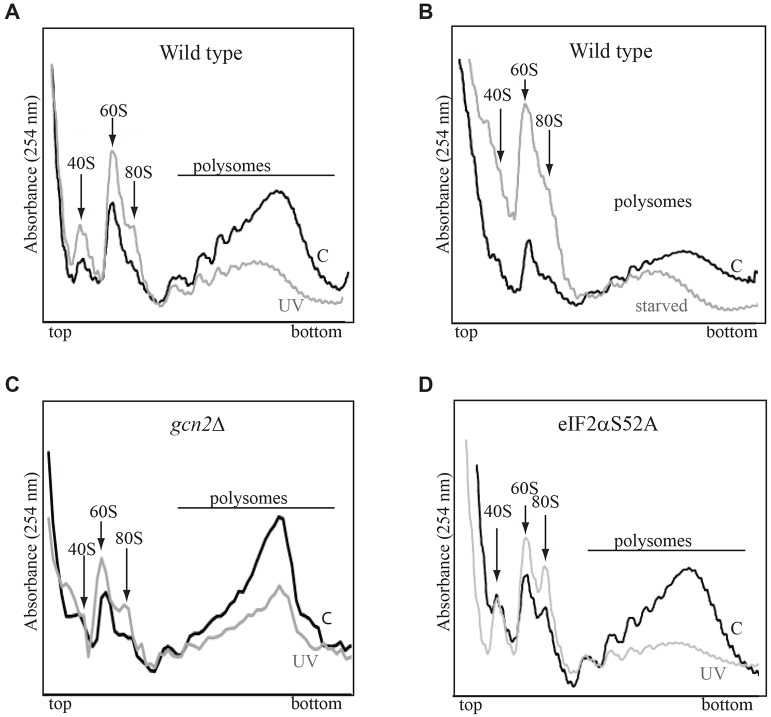
**Polysome analyses after stress treatment.** Polysome profiles were analysed from extracts of wild-type *S. pombe* cells (A,B), or the *gcn2*Δ (C) or eIF2αS52A mutant cells (D). The UV-irradiated samples (light grey, marked UV) were collected 30 min after irradiation, and the starved cells were harvested after 2 h of leucine starvation (marked starved in B). The positions in the gradient of the ribosomal particles (40S, 60S, 80S) are marked as well as the position of the polysomes. ‘C’, control.

It is not clear from the above results at which stage translation is inhibited. To gain some insight into this question, we analyzed the polysomes of fission yeast cells before and after stress exposure. After UV irradiation, the fraction of polysomes was decreased but not abolished (Fig. 6). Because, under these conditions, there is virtually no protein synthesis occurring, the relatively high level of remaining polysomes cannot be very active, and the data indicate that elongation is inhibited and that, in these polysomes, the ribosomes are stalled on the mRNA. A similar conclusion has been reached previously for budding yeast cells that were exposed to oxidative stress (Shenton et al., 2006) – the cells contained a sizeable fraction of intact polysomes, yet protein synthesis was dramatically reduced.

**Fig. 7.**
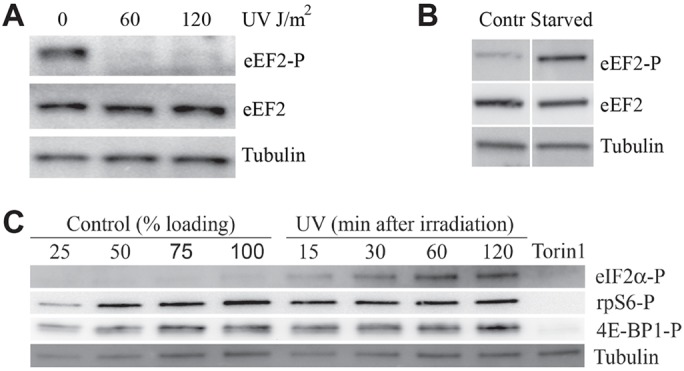
**Changes in EF2 and 4E-BP1 after UV exposure.** GCN2^+/+^ MEF cells were exposed to UV or to serum starvation, and protein extracts were collected for immunoblotting. (A) Phosphorylated EF2 (top, eEF2-P), total EF2 (middle) and γ-tubulin (bottom) were measured in samples collected 30 min after irradiation; the doses of UV are indicated on the top. (B) The cells were starved for serum for 4 h, and the levels of the same proteins as those shown in A were measured. (C) The cells were UV irradiated (60 J/m^2^), and protein samples were collected at different time points after exposure (indicated in minutes). A dilution series (percentages indicated) of extracts from unirradiated control cells were loaded for comparisons (leftmost four lanes). The rightmost lane contains an extract from cells treated with Torin1 for 1 h. The immunoblot was probed with antibodies specific for (from the top) phosphorylated eIF2α, phosphorylated rpS6 (rpS6-P), phosphorylated 4E-BP1 (4E-BP1-P) and γ-tubulin. Contr, control.

Even though initiation of translation is considered to be the most important regulatory step of the translation cycle (Aitken and Lorsch, 2012; Sonenberg and Hinnebusch, 2007, 2009), there is increasing attention on regulatory mechanisms that affect elongation. There is evidence that phosphorylation of the elongation factor eEF2 at residue Y56 in mammalian cells can negatively regulate translation (Leprivier et al., 2013). However, we found that phosphorylation of eEF2 was detectable in unperturbed GCN2^+/+^ MEF cells (Fig. 7A), strongly induced after starvation (Fig. 7B), but could not be detected after UV irradiation (Fig. 7A). This is evidence that the apparent UV-induced inhibition of elongation does not operate through the phosphorylation of eEF2. Furthermore, the phosphorylation site is not conserved in *S. pombe*, making it less likely that this modification is the cause of the translational inhibition observed in fission yeast.

Other candidates that are likely to be responsible for the UV-induced translational inhibition include the Tor kinase, which phosphorylates a number of proteins in eukaryotic cells to regulate translation. The mTOR kinase of mammalian cells phosphorylates the eukaryotic initiation factor 4E-binding protein 1 (4E-BP1), which prevents the initiation factor eIF4E from binding to the translation scaffold protein eIF4G, inhibiting initiation of translation (Proud, 2004). When phosphorylated, 4E-BP1 cannot bind to eIF4E, which is then free to initiate translation. We analysed the phosphorylation status of 4E-BP1 protein at different times after exposure to UV (Fig. 7C). There was no evidence of any reduction in the level of phosphorylated protein. Consistently, we found no change in the level of phosphorylation of the ribosomal protein RPS6, another phosphorylation event that is commonly used as a readout for mTOR activity. Similarly, there is no change in rpS6 phosphorylation after exposure of fission yeast to UV (Rødland et al., 2014), suggesting that the activity of Tor is not changed by UV. Control experiments showed that eIF2α was phosphorylated after UV and that phosphorylation of RPS6 was abolished after the use of the mTOR inhibitor Torin1 (Fig. 7C). We conclude that changes in the level of mTOR activity are not likely to be responsible for the inhibition of protein synthesis after UV exposure.

**Fig. 8.**
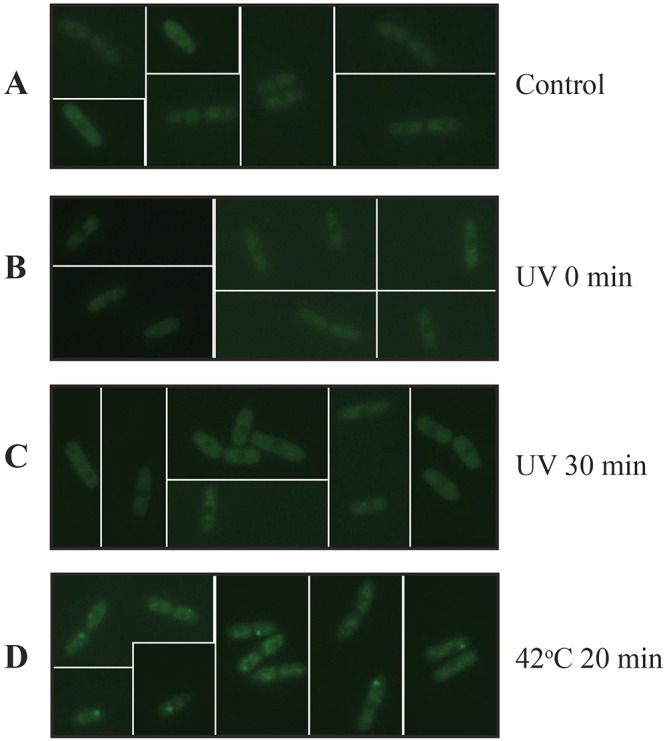
**Stress granules in exponentially growing cells of *S. pombe* strain 489.** A representative collection of pictures of individual cells analysed by using fluorescence microscopy were assembled after no treatment (A), or 0 (B) or 30 (C) min after UV exposure, and after 20 min at 42°C (D).

Many forms of stress induce cytoplasmic RNA granules in fission yeast (Nilsson and Sunnerhagen, 2011) and in budding yeast (Grousl et al., 2009), and it is conceivable that after UV, some of the mRNA is sequestered in such stress-induced granules so that translation is inhibited. To investigate this possibility, we monitored the accumulation of the protein Sum1 in cytoplasmic foci (Dunand-Sauthier et al., 2002) after exposure to UV. We could not detect any sign of stress granule formation in fission yeast cells after a UV dose that abolishes almost all translation (Fig. 8A–C). As a positive control, a large fraction of the cells displayed clearly visible stress granules after 20 min at 42°C (Fig. 8D).

## DISCUSSION

It is the accepted view that, after exposure to stress, phosphorylation of eIF2α leads to a global downregulation of cap-dependent translation. This model builds on the classic observations that, in cells starved for amino acids, eIF2α phosphorylation and global downregulation of translation correlate, and on the fact that phosphorylation of eIF2α prevents eIF2 rejuvenation to the active GTP-bound form. However, it is technically challenging to determine to what extent this downregulation is due to eIF2α phosphorylation or to a lack of amino acids, which in itself is expected to prevent translation.

In contrast to the classic view, we have observed that global stress-induced translational depression after UV is not due to eIF2α phosphorylation. One explanation for our observations could be that the dose of UV seriously damages the ribosomes, or the tRNA, rRNA or mRNA required for translation (Casati and Walbot, 2004; Deng et al., 2002; Iordanov et al., 1998). Such ribotoxic stress and/or crosslinking between ribosomal proteins and RNA can be expected to reduce protein synthesis, and this reduction would occur independently of Gcn2 or eIF2α phosphorylation. We have two strong arguments against this explanation. First, we have shown that in an *in vitro* assay, protein synthesis in extracts of wild-type cells was abolished after UV irradiation of the cells, whereas no inhibition was observed in extracts from a *gcn2*Δ mutant (Tvegård et al., 2007), not even when the dose was doubled or tripled (Grallert and Boye, 2007). Second, there are some proteins that are synthesized at higher rates in UV-irradiated than in unirradiated control cells (Tvegård et al., 2007), strongly suggesting that the translational machinery is intact and largely undamaged by the radiation. If the UV dose used for *S. pombe* is not sufficient to destroy the translation machinery, this is even less likely to happen for the budding yeast or mammalian cells, which received much lower doses of UV. These findings allow us to conclude that although UV certainly has the capacity to damage RNA and proteins, the doses employed here are far below that required to destroy important elements of the translational machinery.

In the present experiments we have used two different stress-inducing agents and three different model organisms, and found basically the same results – translation is severely depressed in a manner independent of Gcn2 and of eIF2α phosphorylation. Our data support the conclusion that global downregulation of translation can be achieved through a pathway that is distinct from that of the classic Gcn2-dependent pathway. This novel pathway is operative after at least two forms of stress, exposure to UV and to H_2_O_2_. Furthermore, it is likely to be universally conserved because it is retained in both the two yeast species, which are widely separated in evolution, and also in mammalian cells.

The role of Gcn2 and eIF2α phosphorylation in regulating protein synthesis after exposure to UV has not been extensively investigated. More specifically, only in a few cases has the translation rate in stressed mammalian cells been measured and only after starving the cells before analysis, which in itself induces phosphorylation of eIF2α. The present data show that even though eIF2α is phosphorylated in mammalian cells after UV, this phosphorylation is only partly responsible for the inhibition of protein synthesis.

A recent, systematic study of translation factors has revealed that elongation factors exert stronger control over protein synthesis rates than initiation factors (Firczuk et al., 2013). Translation elongation is proposed to represent a rich source for the regulation of gene expression (Chu et al., 2013), and several mechanisms to regulate translation elongation have been discovered. All translation elongation factors are phosphoproteins that are modified through kinase-mediated pathways in both mammals (Browne and Proud, 2002) and budding yeast (Patel et al., 2002; Stark et al., 2010). Also, direct modifications of the ribosome subunits are thought to regulate elongation and efficiency of translation (Krieg et al., 1988). In the present work, we do not identify the inhibitory mechanism, but we have excluded the involvement of some prominent candidate proteins, such as eIF2α, eEF2 and mTOR.

Our data support and extend some other indications in the literature (Ashe et al., 2001; Hamanaka et al., 2005; Shenton et al., 2006) that stress-induced translational inhibition might occur through a mechanism that is independent of eIF2α. It is known that eIF2α phosphorylation induces a reprogramming of the selection of mRNAs for translation (Hinnebusch, 2005; Sonenberg and Hinnebusch, 2009; Vattem and Wek, 2004), and we speculate that the main function of eIF2α phosphorylation after UV-irradiation and oxidative stress is to selectively regulate translation of important stress-response proteins rather than to function as a global inhibitor of translation. There is no doubt that phosphorylation of eIF2α contributes to inhibit initiation of translation (Sonenberg and Hinnebusch, 2009) under some conditions. The level of inhibition is most likely to be dependent on the level of phosphorylation – i.e. the fraction of eIF2α molecules that are phosphorylated. Therefore, it is believed that at high levels of phosphorylation, translation is completely blocked at the initiation level. However, the present data strongly indicate that this is not the situation after UV irradiation or oxidative stress, and it is not clear to what extent this is valid for other forms of stress. Importantly, our data show that the presence or absence of phosphorylated eIF2α does not influence the total level of translation after UV (Fig. 1A), arguing that this phosphorylation is not important for global regulation of translation under the present conditions. Therefore, we suggest that the mechanism revealed in this work is the major mechanism to inhibit protein synthesis after many forms of stress. Possibly, the phosphorylation of eIF2α serves to fine-tune the selection of mRNA translation, meaning that certain target mRNAs are translated and not others.

## MATERIALS AND METHODS

### Cells and cell handling

All strains of *S. pombe* used in this study are derivatives of the L972 h- wild-type strain (Leupold, 1950) – wild-type strain 489 (from Paul Nurse, The Francis Crick Institute, London) and derivatives containing *gcn2*Δ or *tif211*-eIF2αS52A:*ura4^+^* (Tvegård et al., 2007). Media and conditions were as described previously (Moreno et al., 1991). Growth was measured using optical density at 595 nm. The cells were grown exponentially at 25°C in liquid Edinburgh minimal medium (EMM) to a cell concentration of about 10^7^/ml (equivalent to an OD of ∼0.5) before the cells were irradiated. The strains of *S. cerevisiae* (wild-type, *gcn2*Δ or *SU12*-S51A, from Tom Dever, National Institutes of Health, Washington, DC) were grown in minimal SD base (catalogue number 630411, Clontech) at 25°C with appropriate supplements and to a cell concentration of about 10^7^/ml [equivalent to an OD (600 nm) of ∼0.45]. MEFs (either GCN^+/+^ or GCN2^−/−^) were cultivated in Dulbecco's modified Eagle's medium (Invitrogen) supplemented with 10% fetal bovine serum (FBS) and 1% penicillin-streptomycin at 37°C in a humidified environment with 5% CO_2_.

### Translation assays

We used a metabolic labelling approach based on incorporation of the noncanonical amino acid l-homopropargylglycine (HPG; Life Technologies) into proteins followed by chemoselective fluorescence tagging by means of ‘click chemistry’ (Best, 2009; Dieterich et al., 2010), using the Click-iT cell reaction buffer kit (Life Technologies). To label newly synthesized proteins, we added 50 µM HPG to the cultures before UV irradiation or H_2_O_2_ treatment, unless otherwise noted. Samples (1 ml) were collected at the indicated time points after treatment, and the cells were fixed in ice-cold 70% ethanol, washed in 0.5 ml PBS, permeabilized in 1% Triton X-100 in 0.5 ml PBS, washed and blocked with 0.5 ml 1% BSA in PBS. ‘Click chemistry’ was performed according to the manufacturer's protocol to join the amino acid alkyne with a fluorochrome azide. Incorporation was measured by using flow cytometry (LSR II flow cytometer, BD Biosciences) and the median fluorescence intensity per cell of Alexa-Fluor-647 azide. Sytox Green and DAPI were used to stain the DNA for flow cytometry and fluorescence microscopy, respectively. Multimers of cells were excluded from the analysis as previously described (Knutsen et al., 2011). Samples without HPG were used as negative controls.

To label newly synthesized proteins in mammalian cells, O-propargyl-puromycin (OPP; Life Technologies) was added immediately after irradiation to a final concentration of 10 µM, and the cells were detached through trypsinisation and fixed in 70% ice-cold ethanol at different times. The cells were washed once in PBS containing 1% FBS before OPP was tagged with Alexa-Fluor-488 in a Click-iT reaction, as described by the manufacturer. Incorporated OPP was measured by using flow cytometry (as described above for *S. pombe*). FxCycle Far Red (Life Technologies) was used to stain DNA, and multimers of cells were excluded. Samples without OPP were used as negative controls.

All translation experiments were repeated at least three times, with qualitatively the same results. Typical results are shown.

### Stress exposure

The appropriate fission yeast mutants were irradiated in a suspension under continuous stirring to distribute the irradiation dose equally to all cells (Nilssen et al., 2003). The standard dose given was equivalent to a surface dose of 1100 J/m^2^, which induces a checkpoint response in the cells, but results in more than 90% cell survival. Cells of *S. cerevisiae* are less resistant to UV and were exposed to 220 J/m^2^ by using the same protocol. Mammalian cells were exposed to UV (60 or 120 J/m^2^) after as much medium as possible had been siphoned off of the culture dishes. The medium was rapidly replaced after irradiation. Oxidative stress was applied to a culture of *S. pombe* cells by adding 5 mM H_2_O_2_ for 15 min, as described (Krohn et al., 2008). For leucine starvation, asynchronous cells auxotrophic for leucine were incubated in EMM without leucine for 2 h (Krohn et al., 2008). MEF cells were starved through incubation in medium without FBS for 4 h.

### Immunoblotting

Total cell extracts of yeast cells for immunoblotting were generated through trichloroacetic acid extraction (Caspari et al., 2000). The extracts were run on polyacrylamide gels, blotted and probed with antibodies against phosphorylated eIF2α (1:2000, Biosource) and α-tubulin (1:30,000, Sigma-Aldrich). Appropriate ECL kits from Amersham Biosciences were used for detection. For mammalian cells, total extracts were made by lysing the cells directly in 2× Laemmli sample buffer. Blots were probed with antibody against eEF2-P (T56) (1:1000, Cell Signaling), phosphorylated eIF2α (as above), 4E-BP1 phosphorylated at T37/T46 (1:1000, Cell Signaling), and phospho-(Ser/Thr) Akt substrate (1:1000, Cell Signaling) for detection of phosphorylated rpS6. Antibodies against γ-tubulin (1:10,000, Sigma-Aldrich), eIF2α (1:500, Santa Cruz Biotechnology) or eEF2 (1:1000, Cell Signaling) were used as controls. Inhibition of mTOR was performed by incubating the cells with Torin1 from Selleckchem (Houston, TX, USA), at a concentration of 100 nM for 1 h (Fig. 7C).

### Polysome profiling

For polysome profiling, cycloheximide was added to the culture to a final concentration of 0.1 mg/ml 30 min after UV irradiation and after 2 h of starvation for leucine. Cell extracts were applied to 10–50% sucrose gradients, centrifuged and analysed as described previously (Swaminathan et al., 2006).
